# Core outcome sets through the healthcare ecosystem: the case of type 2 diabetes mellitus

**DOI:** 10.1186/s13063-020-04403-1

**Published:** 2020-06-25

**Authors:** Susanna Dodd, Nicola Harman, Nichole Taske, Mark Minchin, Toni Tan, Paula R. Williamson

**Affiliations:** 1 Department of Health Data Science, University of Liverpool (a member of Liverpool Health Partners), Liverpool, UK; 2 NICE Centre for Guidelines/Quality and Leadership Programme, Manchester, UK

**Keywords:** Core outcome sets, Electronic health records, Quality indicators, Quality standards, Clinical guidelines

## Abstract

**Background:**

It is increasingly accepted that insufficient attention has been given to the patient health outcomes that are important to measure in comparative effectiveness research that will inform decision-making. The relationship between outcomes chosen for comparative effectiveness research, outcomes used in decision-making in routine care, and outcome data recorded in electronic health records (EHR) is also poorly understood. The COMET Initiative (http://www.comet-initiative.org/. Accessed 3 Apr 2020) supports and encourages the development and use of ‘core outcome sets’ (COS), which represent the minimum set of patient health outcomes that should be measured and reported for a specific condition. There is growing interest in identifying how COS might fit into the different stages of the healthcare research and delivery ecosystem, and whether inclusion in the EHR might facilitate this.

**Methods:**

We sought to determine the degree of overlap between outcomes within COS for research and routine care, EMA, FDA and NICE guidelines, NICE quality statements/indicators, EHR and a point-of-care randomised clinical trial, using type 2 diabetes (T2D) as a case study.

**Results:**

There is substantial agreement about important patient outcomes for T2D for research and healthcare, with associated coverage within the UK general practice EHR.

**Conclusions:**

This case study has demonstrated the potential for efficient research and value-based healthcare when the EHR can include COS for both research and care, where the COS comprises outcomes of importance to all relevant stakeholders. However, this concordance may not hold more generally, as the focus on patient-centred outcomes may well be greater in T2D than in other conditions. Work is ongoing to examine other clinical areas, in order to highlight any current inefficiencies when health outcomes in research and healthcare do not agree with core outcomes identified by patients, clinicians and other key stakeholders.

## Background

Measuring patient health outcomes helps to inform decision-making in healthcare, decisions that are made by patients, healthcare professionals and commissioners/payers. This includes both outcomes chosen for clinical trials designed to inform decision-making, and outcome data that should be collected in routine care to inform decision-making.

The Core Outcome Measures in Effectiveness Trials (COMET) Initiative [1, 2] brings together people interested in the development and application of agreed standardised sets of outcomes, known as “core outcome sets” (COS). One of the successes of COMET has been the development of a publicly available searchable database of completed and ongoing projects in COS development [2–8]. COS may be developed for research or clinical practice, and are determined by consensus amongst health professionals, researchers, policymakers and patients or their representatives, thus ensuring the priorities and expertise of these key stakeholders determine the most important outcomes to measure for a given condition. COS are increasingly being recommended for use by trial funders and healthcare organisations [9].

**Fig. 1:**
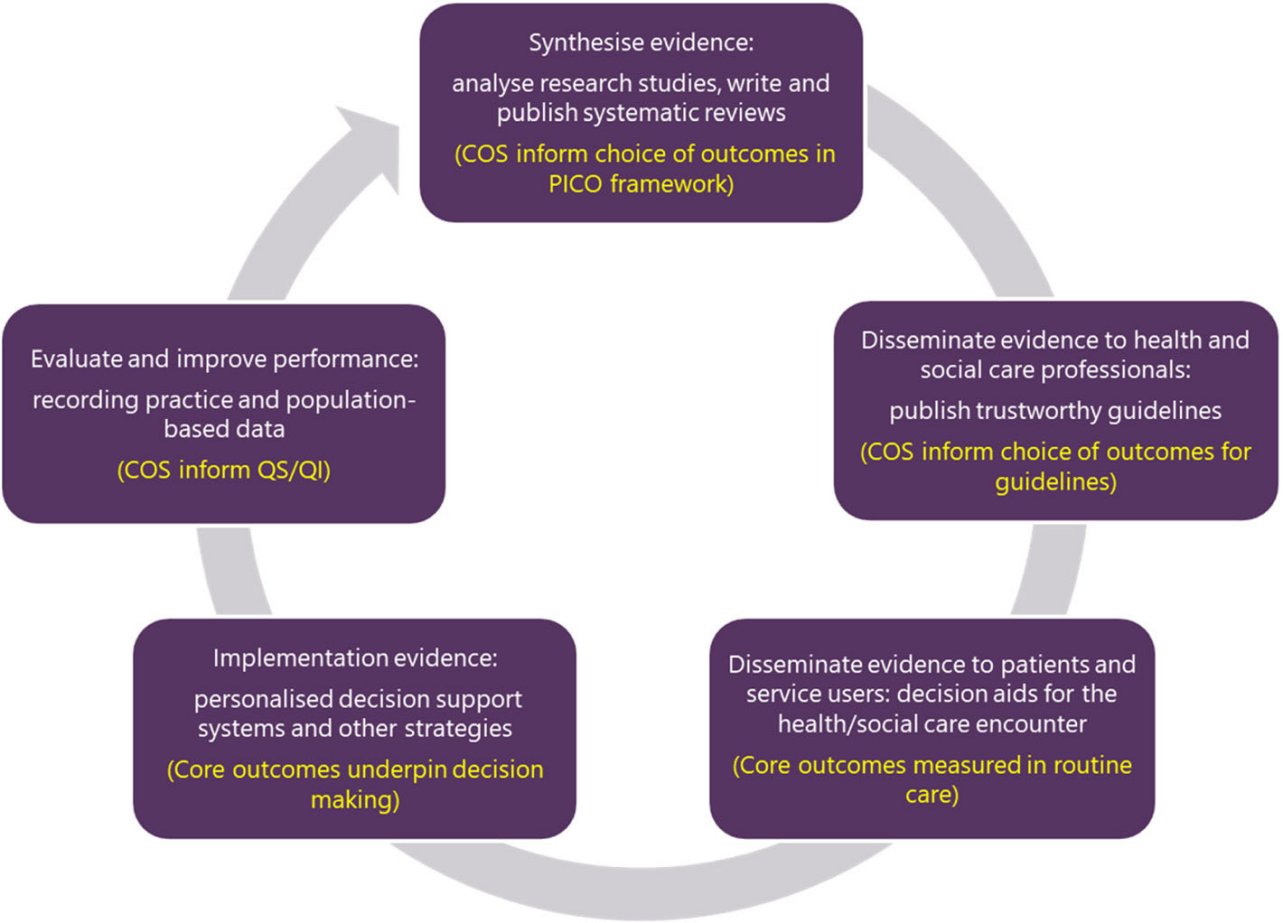
Healthcare research ecosystem (adapted from NICE Connect)

It has been suggested that COS for research may be used to inform systematic reviews, Health Technology Assessments (HTA) and subsequently clinical guidelines, audit and quality standards (QS) [10]. QS set out the most important areas for quality improvement in healthcare, which are in turn used to determine healthcare quality indicators (QIs) [11] (Fig. 1). QI are increasingly being used to monitor the quality of healthcare provision, in order to gauge performance in terms of healthcare structure, processes and patient outcomes [12]. A review of QI studies published up to 2009 found that 69% included patient health outcomes in addition to process and structure outcomes [13]. QI related to patient health outcomes will continue to grow in importance, given the increasing interest in value-based healthcare [14]. Value-based healthcare is defined as “the equitable, sustainable and transparent use of the available resources to achieve better outcomes and experiences for every person” [15].

Organisations that rely on evidence to inform decision-making, such as the UK National Institute for Health and Care Excellence (NICE), or on measurements to support improvement in healthcare services, such as the Healthcare Quality Improvement Partnership (HQIP), are recognising the relevance of considering COS in their work [16, 17]. In 2018, NICE guidance on methods to determine relevant guideline outcomes was updated to indicate that COS should be used, if suitable based on quality and validity [16]. NICE guidelines identify key outcomes (sourcing COS in the COMET database, Cochrane reviews and previous NICE guidelines) during the review protocol stage, which is used by the Guideline Committee to inform the evidence review and then the development of guideline recommendations. These recommendations are then used as a basis for development of NICE QSs. These NICE QSs could be further translated into QIs, which can be used to monitor performance based on the key selected outcomes. In practice, however, the decision by NICE about which outcomes to include within the QI set will largely depend on whether the outcomes are collected in national audits, routine data collections and clinical data within existing electronic health records (EHR).

Clinical trials, evaluations in routine practice settings (including pragmatic trials and routine healthcare audits), monitoring of managed entry/early market access programmes and value-based commissioning (commissioning based on value for patients, clinical expertise and scientific knowledge [18]) could provide more relevant evidence, and be more efficient, if the appropriate health outcomes can be measured, recorded, and extracted from routine data collection systems. There is increased availability of routinely collected data in EHRs (though access to patient level data is strictly controlled in line with information governance restrictions). For example, one of the priorities identified in the UK National Health Service (NHS) Long Term plan that will drive the digital transformation of the NHS in coming years is the linkage of clinical and genomic data collected in routine care and provision of these data for clinical research [19]. The potential of EHRs to facilitate value-based healthcare and comparative effectiveness research by providing a readily available source of data would be greatly enhanced if the outcome data being collected in routine practice and the outcomes chosen for this type of research are aligned.

### Exemplar case study

Type 2 diabetes (T2D) was an opportunistic choice for our exemplar clinical area because the authors were concurrently involved in COS, trial and EHR research activity relating to T2D, namely in the development of the SCORE-IT COS [20] and in the DECIDE trial [21]. The SCORE-IT COS is the only published COS for T2D research, and was the first COS for research to follow the Core Outcome Set-STAndards for Development (COS-STAD) [22] minimum standards, which cover 11 key features of COS development relating to three aspects of the COS development process: scope (including the health condition, population and intervention covered by the COS), stakeholder involvement (including patients, healthcare professionals and researchers) and consensus process (relating to the initial outcomes lists, scoring and consensus decisions, and unambiguous wording of outcomes).

### Aims and objectives

The aim of this case study was to demonstrate proof of concept of COS implementation through the healthcare ecosystem. We sought to determine the concordance between core outcomes included in COS for research for one particular clinical condition and those outcomes included in COS for care; Food and Drug Administration (FDA) and European Medicines Agency (EMA) guidelines; NICE clinical guidelines, QSs and QIs; EHRs (in particular, Clinical Practice Research Datalink [CPRD]); and a pragmatic randomised controlled trial, all relating to the same clinical condition.

## Methods

### COS for research in type 2 diabetes (T2D)

Type 2 diabetes (T2D) was chosen as the exemplar clinical area for this case study because the authors were involved in COS, trial and EHR research activity relating to T2D. First, we identified and extracted the core outcomes from the only published COS for research relating to T2D, SCORE-IT [20].

### COS for routine care in T2D

We then searched for published COS for care relating to T2D, of which there was only one, namely the International Consortium for Health Outcomes Measurement (ICHOM) standard set for type 1 and type 2 diabetes [23]. ICHOM produces COS for routine care, known as “standard sets”, of outcome measures that matter most to patients in order to “unlock the potential of value-based healthcare … to create better value for all stakeholders” [24]. We recorded all core outcomes included in this COS, in order to allow comparison with the core outcomes in SCORE-IT.

### EMA and FDA guidelines in T2D

We then scrutinised regulatory guidelines relating to T2D published by the EMA [25] and the FDA [26], and extracted text relating to each of the SCORE-IT core outcomes from each of these guidelines. We scrutinised the EMA guidance on clinical investigation of products to treat or prevent T2D [25] and the FDA draft guidance document on evaluation of the safety of new drugs for improving glycemic control in T2D [26].

### NICE clinical guidelines, QSs and QIs in T2D

We chose to focus only on UK sources for clinical guidelines, namely those produced by NICE. NICE provides evidence-based recommendations with the aim of improving outcomes for patients using the NHS and other public health and social care services in the UK [27]. We identified NICE [28, 29] guidelines and those in the NICE QS [30] and QI [31] for patients with T2D and extracted all text relating to each of the SCORE-IT core outcomes from each of these sources. The NICE guideline for management of T2D in adults covers care and management [28]. We considered this guideline in combination with the NICE guideline on diabetic foot problems, which covers prevention and management of foot problems caused by diabetes [29]. The NICE QS for diabetes cover care and treatment for adults with type 1 and type 2 diabetes, including prevention, disease management, diabetes-related foot care and diabetes education programmes [30]. The NICE QI relating to diabetes are taken from the NICE list of general practice and clinical commissioning group (payer) indicators [31].

### CPRD (representing EHR)

As with our investigation into core outcomes included within clinical guidelines from UK alone, we decided to focus on EHR sources from the UK only. In the UK, EHR are provided by organisations such as NHS Digital [32], NHSX [33] and CPRD [34], bringing together data from primary care, hospital data and disease-specific databases. NHS Digital collects and publishes data from the health and social care system in England; NHSX (where the “X” stands for “user experience”) oversees the IT strategy across multiple health organisations, including NHS England and the Department of Health and Social Care. CPRD collects routinely collected de-identified data from GP practices across the UK, which can then be linked to other health-related data such as hospital episode statistics (HES). CPRD data cover 45 million patients, 13 million of whom are currently registered. We searched through the list of data field names included in the CPRD database [34] and identified a selection (rather than exhaustive list) of field names that related to each of the SCORE-IT core outcomes. Note that we were assessing consistency in “what” core outcomes are recorded, rather than “how” they are recorded (e.g. ICD code), which we consider would be the next stage of this assessment.

### DECIDE randomised trial

Finally, we identified all the outcomes being collected in an ongoing pragmatic randomised controlled trial in T2D patients (DECIDE [21]) for comparison against the SCORE-IT core outcomes. The DECIDE study is the first point-of-care trial being conducted by CPRD; this trial compares dapagliflozin with usual care in a real-world setting, using three strands of data collection: a study-specific patient portal; EHRs from general practices via two software systems, Vision [35] and EMIS Health (formally known as Egton Medical Information Systems [36]); and linked HES data.

## Results

The mapping of references to each of the SCORE-IT core outcomes within each of the other T2D-related sources (the ICHOM set; FDA, EMA and NICE guidelines; NICE QS and QI; CPRD and the DECIDE trial) is shown in Table 1 (detailed in Supplementary Tables 1a-1d). Note that a tick demonstrates specific correspondence between the wording of the core outcome references in the source compared to the wording in SCORE-IT, whereas a tick in brackets indicates more general alignment (with further detail provided in a footnote) between the wording of text in the source relative to the wording in SCORE-IT.

**Table 1:** Outcomes included in COS for research for T2D (SCORE-IT), COS for routine care (ICHOM set), EMA, FDA and NICE guidelines, NICE QS and QI, CPRD and DECIDE trial

SCORE-IT COS	ICHOM set	Guidelines			NICE QS	NICE QI	CPRD	DECIDE	SCORE-IT core outcome not explicitly mentioned but covered by the following general terms
		EMA	FDA	NICE					
Overall survival	✓						✓	✓	
Death from a diabetes related cause such as heart disease	✓		(✓)^a^	✓			✓	✓	^a^Cardiovascular disease/safety profile
Heart failure	✓	(✓)^ab^	(✓)^ac^	✓	(✓) ^c^	✓	✓		^a^Cardiovascular disease/safety profile ^b^Coronary complications ^c^Diabetes-related complications
Gangrene or amputation of the leg, foot or toe	✓	(✓)^d^	(✓)^c^	✓	✓	✓	✓		^d^Peripheral vascular diseases ^c^Diabetes-related complications
Hyperglycaemic emergencies^1^	✓		(✓)^c^	✓	(✓) ^c^	✓	✓		^c^Diabetes-related complications
Hyperglycaemia			(✓)^c^	✓	(✓) ^c^	✓	✓		^c^Diabetes-related complications
Hypoglycaemia	✓	✓^4^	(✓)^c^	✓	(✓) ^c^	✓	✓	✓	^c^Diabetes-related complications
Cerebrovascular disease	✓	✓	(✓)^c^	✓	(✓)^c^	✓	✓		^c^Diabetes-related complications
Hospital admissions due to diabetes	✓			e	f	✓	✓	✓	^e^ Health economic modelling of the T2D guideline may use estimates on admission rates from sources such as CPRD or HES. ^f^ Mentioned only in relation to type 1 diabetes
Side effects of treatment	✓	✓	✓	✓			✓	✓	
Global quality of life	✓	✓		✓	✓		✓	✓	
Nonfatal myocardial infarction	✓	(✓)^ab^	(✓)^ac^	✓	(✓)^c^	✓	✓		^a^Cardiovascular disease/safety profile ^b^Coronary complications ^c^Diabetes-related complications
Visual deterioration or blindness	✓	(✓)^g^	(✓)^c^	✓	(✓)^c^	✓	✓		^c^Diabetes-related complications ^g^Retinopathy
Glycaemic control	✓	✓^4^	✓	✓	✓	✓	✓	✓	
Neuropathy^2^	✓	✓	(✓)^c^	✓	(✓)^c^	✓	✓		^c^Diabetes-related complications
Kidney function	✓	✓	(✓)^c^	✓	(✓)^c^	✓	✓	✓	^c^Diabetes-related complications
Activities of daily living^3^		✓		h			✓	✓	^h^Activities of daily living are captured in the quality of life measure used by NICE to inform impact on QALYs
Body weight	✓	✓^4^		✓		(✓)^i^	✓	✓	^i^ BMI

*BMI* body mass index, *COS* core outcomes set, *CRPD* Clinical Practice Research Datalink, *EMA* European Medicines Agency, *FDA* U.S. Food and Drug Administration, *HES* Hospital episode statistics, *NICE* National Institute for Health and Care Excellence, *QALY* quality-adjusted life-year, *QI* quality indicator, *QS* quality standard, *T2D* type 2 diabetes

^1^ Including diabetic ketoacidosis and hyperosmolar hyperglycaemic state ^2^ Damage to the nerves caused by high glucose. This can lead to tingling and pain or numbness in the feet or legs. It can also affect bowel control; stomach emptying and sexual function. ^3^ Including those related to personal care; household tasks or community-based tasks. ^4^ Included as core efficacy or safety outcome

### COS for routine care in T2D: ICHOM diabetes set

Sixteen (89%) of the 18 core outcomes included in the SCORE-IT COS were included within the ICHOM diabetes set.

### EMA and FDA guidelines

Thirteen (72%) of the SCORE-IT core outcomes were covered in the FDA guidelines, although 11 of these were referred to in general terms rather than the specific outcome (for example, “adverse cardiovascular events” rather than “heart failure”). The EMA guidance document also included 13 (72%) of the core outcomes (four of these being covered by a general term rather than a specific mention of the core outcome).

### NICE guidelines, QSs and QIs

The NICE guidelines referred to 15 (83%) of the core outcomes, while 12 (67%) and 13 (72%) core outcomes featured in the NICE QS and QI set for T2D respectively (though nine and one of these respectively were covered in general terms rather than specifically mentioning the outcome).

### CPRD and DECIDE trial

All 18 core outcomes were recorded within CPRD, but only 10 (56%) of the SCORE-IT outcomes were included as primary or secondary outcomes in the original DECIDE trial protocol; however, the study team has recognised the SCORE-IT COS and is submitting a protocol amendment to include all outcomes, which is possible because they are routinely collected in primary care.

The two patient-reported outcomes (PROs) in SCORE-IT (global quality of life and activities of daily living) are covered by particular questions or sections within the questionnaires (Hypoglycaemic Fear Survey-II Worry scale, Diabetes Treatment Satisfaction Questionnaire and SF-36 Health Survey v2), which DECIDE patients are requested to complete via the study-specific patient portal.

## Discussion

We believe there is a pressing need to consider the implementation of COS through the healthcare ecosystem in order to facilitate efficient and relevant routine data collection, thereby increasing the usefulness of routine data sources to inform research and the applicability of assessments, audits and value-based commissioning. We have presented a method to determine the degree to which a well-developed COS for research is incorporated across the entire research and healthcare ecosystem.

We have assessed whether core outcomes included within a COS for research align with patient outcomes referred to within a COS for routine care, EMA and FDA regulatory guidelines, and clinical guidelines and corresponding QS and QI, as well as trial outcomes and data fields recorded within EHRs. Although we have focused on one clinical area and considered QS, QI and EHR relevant to UK only, the principles we have adopted are transferable across condition and disease areas and other countries. Note that in this case study we have considered NICE guidelines rather than technology appraisals, as a different approach for determining relevant outcomes is used for NICE appraisals (being typically manufacturer led but influenced by existing appraisals in the same drug class/disease area) than for guidelines. However, appraisals are also increasingly featuring input from COS (for example, those conducted by the SBU in Sweden [37]) and therefore a future extension of this exercise could usefully also determine whether outcomes considered within technology appraisals match those in relevant COS.

Although most COS have been developed separately for research and for care, an increasing number of COS developers are also intending their COS for research to be used in routine care and clinical audit, serving as a thread that pulls all the way through the healthcare system. To date, of 333 published COS across a broad range of clinical areas [8], 35 (11%) are for both research and routine practice. However, of 247 ongoing COS, 131 are for research with the remaining 116 (47%) being developed for both research and routine care.

A review of the 32 published studies where 35 COS are developed for use in both research and routine care indicates that such publications tend to include very little detail regarding the challenge of determining outcome data collection requirements for both settings. There has been considerable research into the barriers and facilitators to routine outcome measurement by health professionals (for example [38]), including the collection of PROs [39–42] and implementation of EHRs in hospitals [43], as well as potential interventions to promote the use of standardised outcome measures [44]. However, in order to make sure that the efforts involved in addressing these challenges are warranted and worthwhile, it is important first to ensure that the outcomes being recorded are of key relevance to important stakeholders. Collection of routine data to monitor healthcare provision should be guided by the priorities of patients and clinicians, thus ensuring that services are audited with respect to the most important outcomes. Identifying these most important outcomes is the aim of COS development.

The EHR also presents a potential platform for capturing PRO directly from patients, for example, if they can be collected via integrated patient portals. Collecting PRO data alongside routinely collected data will enhance the process of audit of quality of care, allowing monitoring of outcomes from the patient perspective [19] which will, in turn, inform subsequent updates of guideline recommendations. There is a growing interest globally for healthcare system and life science industry partners to adopt the principles of value-based healthcare [14] to support sustainability and guide investment decisions. Measuring outcomes that matter to stakeholders, including both patients and clinicians, at scale is fundamental to understanding where the highest value lies.

## Conclusion

In this T2D case study, we found that the COS for research and COS for care are almost identical and largely concur with the outcomes featured in drug regulators’ guidelines, which are in turn reflected in UK clinical guidelines and their associated quality statements and indicators. In the UK, these core outcomes are also collected in the EHR. However, this concordance may not hold more generally, and further work is needed to examine other clinical areas and other national clinical guidelines.

## Supplementary information

**Additional file 1: Supplementary Table 1a.** Outcomes in COS for research for T2D (SCORE-IT), COS for routine care (ICHOM set), NICE QS and QI, CPRD and DECIDE trial. **Supplementary Table 1b.** Outcomes in NICE guidelines. **Supplementary Table 1c.** Outcomes in FDA guidelines. **Supplementary Table 1d.** Outcomes in EMA guidelines.

## Data Availability

Not applicable.

## Abbreviations

COS: Core outcome set
COMET: Core Outcome Measures in Effectiveness Trials
COS-STAD: Core Outcome Set-STAndards for Development
CPRD: Clinical Practice Research Datalink
EHR: Electronic health record
EMA: European Medicines Agency
FDA: Food and Drug Administration
HES: Hospital episode statistics
HTA: Health Technology Assessment
HQIP: Healthcare Quality Improvement Partnership
ICHOM: International Consortium for Health Outcomes Measurement
NHS: National Health Service
NICE: The National Institute for Health and Care Excellence
PRO: Patient-reported outcome
QS: Quality standard
QI: Quality indicator
T2D: Type 2 diabetes
