# Supplementary material for: Core outcome sets through the healthcare ecosystem: the case of type 2 diabetes mellitus
Source: Trials. 2020 Jun 25;21:570. doi: 10.1186/s13063-020-04403-1 (PMC7318375; doi:10.1186/s13063-020-04403-1)
Supplement: Supplementary file 1 — Additional file 1: Supplementary Table 1a. Outcomes in COS for research for T2D (SCORE-IT), COS for routine care (ICHOM set), NICE QS and QI, CPRD and DECIDE trial. Supplementary Table 1b. Outcomes in NICE guidelines. Supplementary Table 1c. Outcomes in FDA guidelines. Supplementary Table 1d. Outcomes in EMA guidelines. [file 13063_2020_4403_MOESM1_ESM.zip › Supplementary Table 1aR1.pdf]

Supplementary Table 1a. Outcomes in COS for research for type 2 diabetes (SCORE-IT), COS for routine care (ICHOM set), NICE QS and QI, CPRD and DECIDE trial

SCORE-IT COS	ICHOM set	NICE QS	NICE QI	CPRD	DECIDE
Overall survival	Vital status			Yes (Certificate – death)	Serious adverse events
Death from a diabetes related cause such as heart disease	Cause of death			Yes (SD18 - cause of death clarif)	Serious adverse events
Heart failure	Chronic Heart Failure	**QS4:** Incidence of diabetes-related complications	**CCG61** Admission rates due to heart failure in people with diabetes.<br>**CCG16** Proportion of adults with diabetes with a HES recording of National Diabetes Audit complications	Yes (Heart failure confirmed)	
Gangrene or amputation of the leg, foot or toe	Lower Limb Amputation	**QS4:** Incidence of diabetes-related complications<br>**QS5/6:** Incidence of foot and lower limb amputations in people with diabetes	**CCG65** Admission rates due to lower limb amputations in people with diabetes.	Yes (Gangrene; Type II diabetes mellitus with gangrene)	
Hyperglycaemic emergencies (to include diabetic ketoacidosis and hyperosmolar hyperglycaemic state)	Hyperosmolar hyperglycaemic syndrome Diabetic ketoacidosis	**QS4:** Incidence of diabetes-related complications	**CCG15** The proportion of adults with diabetes who have an emergency admission for diabetic ketoacidosis.<br>**CCG64** Admission rates due to diabetic ketoacidosis in people with diabetes.<br>**CCG16** Proportion of adults with diabetes with a HES recording of National Diabetes Audit complications	Yes (Diabetes mellitus NOS with ketoacidosis; Other specified diabetes mellitus with ketoacidosis)	
Hyperglycaemia		**QS4:** Incidence of diabetes-related complications	**CCG16** Proportion of adults with diabetes with a HES recording of	Yes (Hyperglycaemia)	

SCORE-IT COS	ICHOM set	NICE QS	NICE QI	CPRD	DECIDE
			National Diabetes Audit complications		
Hypoglycaemia	Hypoglycaemia	**QS4:** Incidence of diabetes-related complications	**CCG16** Proportion of adults with diabetes with a HES recording of National Diabetes Audit complications	Yes (Hypoglycaemia unspecified)	1) No reported severe or documented hypoglycaemic events since randomisation<br>2) Number of contacts due to hypoglycaemic events
Cerebrovascular disease (including stroke. subarachnoid haemorrhage. transient ischaemic attack and vascular dementia)	Micro- and macrovascular complications:<br>Cerebrovascular Disease<br>Acute Cardiovascular Events (Stroke and Myocardial Infarction)<br>Peripheral Artery Disease<br>Ischemic Heart Disease	**QS4:** Incidence of diabetes-related complications	**CCG12** The proportion of adults with a diagnosis of diabetes who have a recording of MI, stroke and end stage kidney disease.<br>**CCG62** Admission rates due to stroke in people with diabetes.<br>**CCG16** Proportion of adults with diabetes with a HES recording of National Diabetes Audit complications	Yes (Stroke due to intracerebral haemorrhage; Subarachnoid haemorrhage NOS; Suspected transient ischaemic attack; Vascular dementia)	
Hospital admissions due to diabetes	Healthcare utilization (Hospitalization, Emergency Room Utilization)	***QS7:** Length of hospital stay (mentioned only in relation to type 1 diabetes)*	**CCG58** Admission rates for people with diabetes due to complications associated with diabetes.<br>**CCG60** Admission rates due to myocardial infarction in people with diabetes.<br>**CCG61** Admission rates due to heart failure in people with diabetes.<br>**CCG62** Admission rates due to stroke in people with diabetes.	Yes (H/O: Admission in last year for diabetes foot problem)	Number of hospitalizations

SCORE-IT COS	ICHOM set	NICE QS	NICE QI	CPRD	DECIDE
			**CCG63** Admissions rates for renal replacement therapy in people with diabetes.<br>**CCG64** Admission rates due to diabetic ketoacidosis in people with diabetes.<br>**CCG65** Admission rates due to lower limb amputations in people with diabetes.		
Side effects of treatment	Treatment complications (Lipodystrophy under Injection site complications)			Yes (Has shown side effects from medication)	Proportions of patients discontinued from study medication due to serious adverse events.<br>Marked laboratory abnormalities during treatment
Global quality of life (including physical. mental. and social wellbeing)	Psychological wellbeing<br>Diabetes distress<br>Depression	**QS2:** Patient satisfaction with ability to self-manage their type 2 diabetes after attending a structured education programme		Yes (Quality of life scale)	1) Hypoglycaemic Fear Survey-II Worry scale<br>2) Diabetes Treatment Satisfaction Questionnaire<br>3) SF36v2
Nonfatal myocardial infarction	Acute Cardiovascular Events (Stroke and Myocardial Infarction)	**QS4:** Incidence of diabetes-related complications	**CCG12** The proportion of adults with a diagnosis of diabetes who have a recording of MI, stroke and end stage kidney disease.<br>**CCG60** Admission rates due to myocardial infarction in people with diabetes.<br>**CCG16** Proportion of adults with diabetes with a HES recording of	Yes (MI - acute myocardial infarction)	

SCORE-IT COS	ICHOM set	NICE QS	NICE QI	CPRD	DECIDE
			National Diabetes Audit complications		
Visual deterioration or blindness	Vision	**QS4:** Incidence of diabetes-related complications	**NM98** The percentage of patients with diabetes, on the register, who have a record of retinal screening in the preceding 12 months.<br>**CCG16** Proportion of adults with diabetes with a HES recording of National Diabetes Audit complications	Yes (Impaired vision due to diabetic retinopathy)	
Glycaemic control	Glycaemic control	**QS4:** Proportion of adults with type 2 diabetes who are started on dual therapy when their HbA1c level is 58 mmol/mol (7.5%) or above after 6 months with single-drug treatment<br>**QS4:** Adults with type 2 diabetes feel supported to aim for an HbA1c level of 53 mmol/mol (7.0%) or less	**NM97** The percentage of patients with diabetes, on the register, in whom the last IFCC-HbA1c is 75 mmol/mol or less in the preceding 12 months.<br>(**NM96** 64 mmol/mol or less)<br>(**NM141** 58 mmol/mol or less)<br>(**NM157** 58mmol/mol or less) in people without moderate or severe frailty)<br>(**NM158** 75 mmol/mol or less) in people with moderate or severe frailty))<br>**NM74** The percentage of patients with diabetes who have had the following care processes performed in	Yes (HbA1c level (DCCT aligned))	1) HbA1c reduction vs. baseline (≥ 0.5%)<br>2) Change from baseline in HbA1c

SCORE-IT COS	ICHOM set	NICE QS	NICE QI	CPRD	DECIDE
			the preceding 12 months: including<br>• HbA1c measurement.		
Neuropathy (damage to the nerves caused by high glucose. This can lead to tingling and pain or numbness in the feet or legs. It can also affect bowel control; stomach emptying and sexual function)	Nervous system complications (Autonomic Neuropathy,<br>Peripheral Neuropathy, Charcot's Foot, Erectile Dysfunction, Lower Limb Ulcers)	**QS4:** Incidence of diabetes-related complications	**NM131** The percentage of patients with diabetes with a record of testing of foot sensation using a 10g monofilament within the preceding 12 months.<br>**NM13** The percentage of patients with diabetes with a record of a foot examination and risk classification: 1) low risk (normal sensation, palpable pulses), 2) increased risk (neuropathy or absent pulses), 3) high risk (neuropathy or absent pulses plus deformity or skin changes or previous ulcer) or 4) ulcerated foot within the preceding 15 months.<br>**NM51** The percentage of male patients with diabetes with a record of being asked about erectile dysfunction in the preceding 15 months.<br>**NM52** The percentage of male patients with diabetes who have a	Yes (Diabetic neuropathy)	

SCORE-IT COS	ICHOM set	NICE QS	NICE QI	CPRD	DECIDE
			record of erectile dysfunction with a record of advice and assessment of contributory factors and treatment options in the preceding 15 months.<br>**NM74** The percentage of patients with diabetes who have had the following care processes performed in the preceding 12 months: including<br>• Foot examination.<br>**CCG16** Proportion of adults with diabetes with a HES recording of National Diabetes Audit complications		
Kidney function	Chronic Kidney Disease and Dialysis	**QS4:** Incidence of diabetes-related complications	**NM59** The percentage of patients with diabetes who have a record of an albumin:creatinine ratio (ACR) test in the preceding 15 months.<br>**NM74** The percentage of patients with diabetes who have had the following care processes performed in the preceding 12 months: including<br>• Albumin: creatinine ratio<br>• Serum creatinine measurement.	Yes (Kidney function tests)	Change from baseline in eGFR (ml/min)

SCORE-IT COS	ICHOM set	NICE QS	NICE QI	CPRD	DECIDE
			**NM95** The percentage of patients with diabetes, on the register, with a diagnosis of nephropathy (clinical proteinuria) or micro-albuminuria who are currently treated with an ACE-I (or ARBs)<br>**CCG12** The proportion of adults with a diagnosis of diabetes who have a recording of MI, stroke and end stage kidney disease.<br>**CCG63** Admissions rates for renal replacement therapy in people with diabetes.<br>**CCG16** Proportion of adults with diabetes with a HES recording of National Diabetes Audit complications		
Activities of daily living (including those related to personal care; household tasks or community based tasks)				Yes (Performance test of activities of daily living)	1) Diabetes Treatment Satisfaction Questionnaire<br>2) SF36v2
Body weight	Weight (to determine body mass index in Intermediate Outcomes)		**NM74** The percentage of patients with diabetes who have had the following care processes performed in the preceding 12 months: including<br>• BMI measurement	Yes (Weight monitoring)	1) Weight loss vs. baseline (≥ 2 Kg)<br>2) Change from baseline in total body weight
