# Supplementary material for: Core outcome sets through the healthcare ecosystem: the case of type 2 diabetes mellitus
Source: Trials. 2020 Jun 25;21:570. doi: 10.1186/s13063-020-04403-1 (PMC7318375; doi:10.1186/s13063-020-04403-1)
Supplement: Supplementary file 1 — Additional file 1: Supplementary Table 1a. Outcomes in COS for research for T2D (SCORE-IT), COS for routine care (ICHOM set), NICE QS and QI, CPRD and DECIDE trial. Supplementary Table 1b. Outcomes in NICE guidelines. Supplementary Table 1c. Outcomes in FDA guidelines. Supplementary Table 1d. Outcomes in EMA guidelines. [file 13063_2020_4403_MOESM1_ESM.zip › Supplementary Table 1bR1.pdf]

Supplementary Table 1b. Outcomes in NICE guidelines

SCORE-IT COS	NICE guideline (text taken from "Key priorities for implementation", "Recommendations" and "Research Recommendations")
Overall survival	
Death from a diabetes related cause such as heart disease	2.1 There is limited understanding of the short- and long-term effects of stopping a therapy and switching to another in terms of diabetes control (HbA1c levels), hypoglycaemic risk, weight gain, and cardiovascular morbidity and mortality.
Heart failure	**1.6.24** In adults with type 2 diabetes, do not offer or continue pioglitazone[4] if they have any of the following: heart failure or history of heart failure, hepatic impairment, diabetic ketoacidosis, current, or a history of, bladder cancer, uninvestigated macroscopic haematuria.<br><br>**Footnote [9]** Medicines and Healthcare products Regulatory Agency (MHRA) guidance (2011) notes that cases of cardiac failure have been reported when pioglitazone was used in combination with insulin, especially in patients with risk factors for the development of cardiac failure. It advises that if the combination is used, people should be observed for signs and symptoms of heart failure, weight gain, and oedema. Pioglitazone should be discontinued if any deterioration in cardiac status occurs.<br><br>2.1 There is limited understanding of the short- and long-term effects of stopping a therapy and switching to another in terms of diabetes control (HbA1c levels), hypoglycaemic risk, weight gain, and cardiovascular morbidity and mortality.<br><br>2.2 Randomised controlled trials are therefore needed to better understand the treatment choices that are available which improve blood glucose control and long-term risks of complications associated with diabetes.<br><br>2.4 There is limited evidence in relation to the long-term effects (at least 5 years) of blood glucose lowering therapies, particularly newer agents in terms of efficacy and adverse events (for example, cardiovascular outcomes).
Gangrene or amputation of the leg, foot or toe	**Diabetic foot problems 1.7.11** For guidance on preventing and managing foot problems in adults with type 2 diabetes, see the NICE guideline on diabetic foot problems.<br><br>**Assessing the risk of developing a diabetic foot problem**<br>1.3.3 For adults with diabetes, assess their risk of developing a diabetic foot problem at the following times…<br>1.3.4 When examining the feet of a person with diabetes, remove their shoes, socks, bandages and dressings, and examine both feet for evidence of the following risk factors: including Gangrene.<br>1.3.6 Assess the person's current risk of developing a diabetic foot problem or needing an amputation using the following risk stratification<br>1.4.1 Examples of limb-threatening and life-threatening diabetic foot problems include the following: Gangrene (with or without ulceration).<br><br>**Footnote [5]** Medicines and Healthcare products Regulatory Agency (MHRA) guidance (2017) warned that canagliflozin may increase the risk of lower-limb amputation (mainly toes) in people with type 2 diabetes. At the time of publication (March 2017) evidence did not show an increased risk for dapagliflozin and empagliflozin, but the MHRA advised that the risk may be a class effect.<br><br>2.2 Randomised controlled trials are therefore needed to better understand the treatment choices that are available which improve blood glucose control and long-term risks of complications associated with diabetes.
Hyperglycaemic emergencies (to include diabetic ketoacidosis and hyperosmolar hyperglycaemic state)	**Footnote [6]** Medicines and Healthcare products Regulatory Agency (MHRA) guidance (2016) warned that serious, life-threatening, and fatal cases of diabetic ketoacidosis have been reported rarely in people taking an SGLT-2 inhibitor (a substantial proportion of the cases concerned off-label use in people with type 1 diabetes, which is not recommended). In several cases, blood glucose levels were only moderately elevated. The MHRA advised that

SCORE-IT COS	NICE guideline (text taken from “Key priorities for implementation”, “Recommendations” and “Research Recommendations”)
	healthcare professionals should test for raised ketones in people with ketoacidosis symptoms who are receiving an SGLT-2 inhibitor, even if their plasma glucose levels are near-normal.<br><br>**1.6.24** In adults with type 2 diabetes, do not offer or continue pioglitazone[4] if they have any of the following: heart failure or history of heart failure, hepatic impairment, diabetic ketoacidosis, current, or a history of, bladder cancer, uninvestigated macroscopic haematuria.<br><br>2.2 Randomised controlled trials are therefore needed to better understand the treatment choices that are available which improve blood glucose control and long-term risks of complications associated with diabetes.
Hyperglycaemia	**1.6.15** Be aware that adults with type 2 diabetes who have acute intercurrent illness are at risk of worsening hyperglycaemia. Review treatment as necessary.<br><br>**Rescue therapy at any phase of treatment 1.6.18** If an adult with type 2 diabetes is symptomatically hyperglycaemic, consider insulin (see recommendations 1.6.32–1.6.34) or a sulfonylurea, and review treatment when blood glucose control has been achieved.
Hypoglycaemia	1.6.5 Involve adults with type 2 diabetes in decisions about their individual HbA1c target. Encourage them to achieve the target and maintain it unless any resulting adverse effects (including hypoglycaemia), or their efforts to achieve their target, impair their quality of life.<br><br>**1.6.14** Consider short-term self-monitoring of blood glucose levels in adults with type 2 diabetes (and review treatment as necessary): when starting treatment with oral or intravenous corticosteroids or to confirm suspected hypoglycaemia.<br><br>Blood glucose management: Involve adults with type 2 diabetes in decisions about their individual HbA1c target. Encourage them to achieve the target and maintain it unless any resulting adverse effects (including hypoglycaemia), or their efforts to achieve their target, impair their quality of life.<br>Do not routinely offer self-monitoring of blood glucose levels for adults with type 2 diabetes unless: the person is on insulin or there is evidence of hypoglycaemic episodes or the person is on oral medication that may increase their risk of hypoglycaemia while driving or operating machinery or the person is pregnant, or is planning to become pregnant.<br><br>**1.3.6**: Individualise recommendations for carbohydrate and alcohol intake, and meal patterns. Reducing the risk of hypoglycaemia should be a particular aim for a person using insulin or an insulin secretagogue.<br><br>**1.6.32** When starting insulin therapy in adults with type 2 diabetes, use a structured programme employing active insulin dose titration that encompasses… management of hypoglycaemia, management of acute changes in plasma glucose control…<br><br>**1.6.34** Consider, as an alternative to NPH insulin, using insulin detemir or insulin glargine[10] if… the person's lifestyle is restricted by recurrent symptomatic hypoglycaemic episodes… Consider pre-mixed (biphasic) preparations that include short-acting insulin analogues, rather than pre-mixed (biphasic) preparations that include short-acting human insulin preparations, if… hypoglycaemia is a problem…<br><br>2.1 There is limited understanding of the short- and long-term effects of stopping a therapy and switching to another in terms of diabetes control (HbA1c levels), hypoglycaemic risk, weight gain, and cardiovascular morbidity and mortality.
Cerebrovascular disease (including stroke. subarachnoid haemorrhage. transient ischaemic attack and vascular dementia)	Blood pressure management: Add medications if lifestyle advice does not reduce blood pressure to below 140/80 mmHg (below 130/80 mmHg if there is kidney, eye or cerebrovascular damage). Monitor blood pressure every 1–2 months, and intensify therapy if the person is already on antihypertensive drug treatment, until the blood pressure is consistently below 140/80 mmHg (below 130/80 mmHg if there is kidney, eye or cerebrovascular damage).<br><br>**1.4.3**: Repeat blood pressure measurements within …2 months if blood pressure is higher than 130/80 mmHg and there is kidney, eye or cerebrovascular damage.

SCORE-IT COS	NICE guideline (text taken from "Key priorities for implementation", "Recommendations" and "Research Recommendations")
	**1.4.4**: Provide lifestyle advice (see section 1.3 in this guideline and the lifestyle interventions section in hypertension in adults [NICE guideline CG127]) if blood pressure is confirmed as being consistently above 140/80 mmHg (or above 130/80 mmHg if there is kidney, eye or cerebrovascular damage).<br><br>Similar conditional statement (…if there is kidney, eye or cerebrovascular damage) in **1.4.5 1.4.6**<br><br>2.2 Randomised controlled trials are therefore needed to better understand the treatment choices that are available which improve blood glucose control and long-term risks of complications associated with diabetes.
Hospital admissions due to diabetes	
Side effects of treatment	Blood glucose management: Involve adults with type 2 diabetes in decisions about their individual HbA1c target. Encourage them to achieve the target and maintain it unless any resulting adverse effects (including hypoglycaemia), or their efforts to achieve their target, impair their quality of life.<br><br>Drug treatment: In adults with type 2 diabetes, if metformin is contraindicated or not tolerated, consider initial drug treatment with DPP-4 inhibitor, pioglitazone or a sulfonylurea.<br><br>Be aware that, if metformin is contraindicated or not tolerated, repaglinide is both clinically effective and cost effective in adults with type 2 diabetes.<br><br>When prescribing pioglitazone, exercise particular caution if the person is at high risk of the adverse effects of the drug. Pioglitazone is associated with an increased risk of heart failure, bladder cancer and bone fracture.<br><br>**1.1.1** Adopt an individualised approach to diabetes care that is tailored to the needs and circumstances of adults with type 2 diabetes, taking into account their personal preferences, comorbidities, risks from polypharmacy, and their ability to benefit from long-term interventions because of reduced life expectancy. Such an approach is especially important in the context of multimorbidity.<br><br>**1.6.5** Involve adults with type 2 diabetes in decisions about their individual HbA1c target. Encourage them to achieve the target and maintain it unless any resulting adverse effects (including hypoglycaemia), or their efforts to achieve their target, impair their quality of life.<br><br>**1.6.17** For adults with type 2 diabetes, discuss the benefits and risks of drug treatment, and the options available. Base the choice of drug treatment(s) on:<br>• the effectiveness of the drug treatment(s) in terms of metabolic response<br>• safety (see Medicines and Healthcare products Regulatory Agency [MHRA] guidance) and tolerability of the drug treatment(s)<br>• the person's individual clinical circumstances, for example, comorbidities, risks from polypharmacy<br><br>**1.6.20** Gradually increase the dose of standard-release metformin over several weeks to minimise the risk of gastrointestinal side effects in adults with type 2 diabetes.<br><br>**1.6.21** If an adult with type 2 diabetes experiences gastrointestinal side effects with standard-release metformin, consider a trial of modified-release metformin.<br><br>**1.6.30** In adults with type 2 diabetes, if metformin is contraindicated or not tolerated, and if dual therapy with 2 oral drugs (see recommendation 1.6.26) has not continued to control HbA1c to below the person's individually agreed threshold for intensification, consider insulin-based treatment

SCORE-IT COS	NICE guideline (text taken from “Key priorities for implementation”, “Recommendations” and “Research Recommendations”)
	**1.7.8** When using tricyclic drugs and antihypertensive drug treatments in adults with type 2 diabetes who have autonomic neuropathy, be aware of the increased likelihood of side effects such as orthostatic hypotension.<br><br>2.1 The current practice of 'stopping rules' is typically motivated by either inadequate blood glucose control (rising HbA1c levels) or intolerable side effects.<br><br>2.4 There is limited evidence in relation to the long-term effects (at least 5 years) of blood glucose lowering therapies, particularly newer agents in terms of efficacy and adverse events (for example, cardiovascular outcomes).
Global quality of life (including physical. mental. and social wellbeing)	Blood glucose management: Involve adults with type 2 diabetes in decisions about their individual HbA1c target. Encourage them to achieve the target and maintain it unless any resulting adverse effects (including hypoglycaemia), or their efforts to achieve their target, impair their quality of life.<br><br>1.3.2 Provide dietary advice in a form sensitive to the person's needs, culture and beliefs, being sensitive to their willingness to change and the effects on their quality of life.<br><br>1.6.16 If adults with type 2 diabetes are self-monitoring their blood glucose levels, carry out a structured assessment at least annually. The assessment should include:<br>• the person's self-monitoring skills<br>• the quality and frequency of testing<br>• checking that the person knows how to interpret the blood glucose results and what action to take<br>• the impact on the person's quality of life<br>• the continued benefit to the person<br>• the equipment used.
Nonfatal myocardial infarction	**1.5.2** For guidance on the primary and secondary prevention of cardiovascular disease in adults with type 2 diabetes, see the NICE guidelines on cardiovascular disease and myocardial infarction.<br><br>2.1 There is limited understanding of the short- and long-term effects of stopping a therapy and switching to another in terms of diabetes control (HbA1c levels), hypoglycaemic risk, weight gain, and cardiovascular morbidity and mortality.<br><br>2.4 There is limited evidence in relation to the long-term effects (at least 5 years) of blood glucose lowering therapies, particularly newer agents in terms of efficacy and adverse events (for example, cardiovascular outcomes).<br><br>2.2 Randomised controlled trials are therefore needed to better understand the treatment choices that are available which improve blood glucose control and long-term risks of complications associated with diabetes.
Visual deterioration or blindness	Blood pressure management: Add medications if lifestyle advice does not reduce blood pressure to below 140/80 mmHg (below 130/80 mmHg if there is kidney, eye or cerebrovascular damage). Monitor blood pressure every 1–2 months, and intensify therapy if the person is already on antihypertensive drug treatment, until the blood pressure is consistently below 140/80 mmHg (below 130/80 mmHg if there is kidney, eye or cerebrovascular damage).<br><br>**1.1.2** Take into account any disabilities, including visual impairment, when planning and delivering care for adults with type 2 diabetes.<br><br>1.4.2 Make changes only if there is poor control or if current drug treatment is not appropriate because of microvascular complications or metabolic problems.<br><br>**1.4.3**: Repeat blood pressure measurements within …2 months if blood pressure is higher than 130/80 mmHg and there is kidney, eye or cerebrovascular damage.

SCORE-IT COS	NICE guideline (text taken from “Key priorities for implementation”, “Recommendations” and “Research Recommendations”)
	**1.4.4**: Provide lifestyle advice (see section 1.3 in this guideline and the lifestyle interventions section in hypertension in adults [NICE guideline CG127]) if blood pressure is confirmed as being consistently above 140/80 mmHg (or above 130/80 mmHg if there is kidney, eye or cerebrovascular damage).<br><br>Similar conditional statement (…if there is kidney, eye or cerebrovascular damage) in **1.4.5 1.4.6**<br><br>**Eye disease 1.7.17** On diagnosis, GPs should immediately refer adults with type 2 diabetes to the local eye screening service. Perform screening as soon as possible and no later than 3 months from referral. Arrange repeat structured eye screening annually.<br><br>Similarly for **1.7.18-1.7.25**<br><br>2.2 Randomised controlled trials are therefore needed to better understand the treatment choices that are available which improve blood glucose control and long-term risks of complications associated with diabetes.
Glycaemic control	**1.6 Blood glucose management (covered in 1.6.1-1.6.10: Measurement 1.6.1-1.6.4; Targets 1.6.4-1.6.10)**<br><br>**1.6.1** In adults with type 2 diabetes, measure HbA1c levels at: 3–6-monthly intervals (tailored to individual needs), until the HbA1c is stable on unchanging therapy; 6-monthly intervals once the HbA1c level and blood glucose lowering therapy are stable.<br><br>**1.6.2** Use methods to measure HbA1c that have been calibrated according to International Federation of Clinical Chemistry (IFCC) standardisation.<br><br>**1.6.3** If HbA1c monitoring is invalid because of disturbed erythrocyte turnover or abnormal haemoglobin type, estimate trends in blood glucose control using one of the following: quality-controlled plasma glucose profiles, total glycated haemoglobin estimation (if abnormal haemoglobins), fructosamine estimation.<br><br>**1.6.4** Investigate unexplained discrepancies between HbA1c and other glucose measurements. Seek advice from a team with specialist expertise in diabetes or clinical biochemistry.<br><br>Similarly for **Targets 1.6.4-1.6.10**<br><br>**1.6.25** In adults with type 2 diabetes, if initial drug treatment with metformin has not continued to control HbA1c to below the person's individually agreed threshold for intensification, consider dual therapy…<br><br>Similarly for **1.6.26-1.6.27**<br><br>**1.6.29** Only continue GLP-1 mimetic therapy if the person with type 2 diabetes has had a beneficial metabolic response (a reduction of at least 11 mmol/mol [1.0%] in HbA1c and a weight loss of at least 3% of initial body weight in 6 months).<br><br>**1.6.30** In adults with type 2 diabetes, if metformin is contraindicated or not tolerated, and if dual therapy with 2 oral drugs (see recommendation 1.6.26) has not continued to control HbA1c to below the person's individually agreed threshold for intensification, consider insulin-based treatment<br><br>**1.6.32** When starting insulin therapy in adults with type 2 diabetes, use a structured programme employing active insulin dose titration that encompasses… management of hypoglycaemia, management of acute changes in plasma glucose control…<br><br>2.1 2.1 The current practice of 'stopping rules' is typically motivated by either inadequate blood glucose control (rising HbA1c levels) or intolerable side effects.There is limited

SCORE-IT COS	NICE guideline (text taken from “Key priorities for implementation”, “Recommendations” and “Research Recommendations”)
	understanding of the short- and long-term effects of stopping a therapy and switching to another in terms of diabetes control (HbA1c levels), hypoglycaemic risk, weight gain, and cardiovascular morbidity and mortality<br><br>2.2 Randomised controlled trials are therefore needed to better understand the treatment choices that are available which improve blood glucose control and long-term risks of complications associated with diabetes.<br><br>2.3 Randomised controlled trials are needed to improve understanding of alternative treatment options for adults at second intensification whose blood glucose is inadequately controlled with insulin and/or triple non-insulin-based drug therapies.
Neuropathy (damage to the nerves caused by high glucose. This can lead to tingling and pain or numbness in the feet or legs. It can also affect bowel control; stomach emptying and sexual function)	1.4.2 Make changes only if there is poor control or if current drug treatment is not appropriate because of microvascular complications or metabolic problems.<br><br>**Painful diabetic neuropathy: 1.7.5** For guidance on managing painful diabetic peripheral neuropathy in adults with type 2 diabetes, see the NICE guideline on neuropathic pain in adults.<br><br>**Autonomic neuropathy:**<br>**1.7.6** Think about the possibility of contributory sympathetic nervous system damage for adults with type 2 diabetes who lose the warning signs of hypoglycaemia.<br><br>1.7.7 Think about the possibility of autonomic neuropathy affecting the gut in adults with type 2 diabetes who have unexplained diarrhoea that happens particularly at night.<br><br>1.7.8 When using tricyclic drugs and antihypertensive drug treatments in adults with type 2 diabetes who have autonomic neuropathy, be aware of the increased likelihood of side effects such as orthostatic hypotension.<br><br>1.7.9 Investigate the possibility of autonomic neuropathy affecting the bladder in adults with type 2 diabetes who have unexplained bladder-emptying problems.<br><br>1.7.10 In managing autonomic neuropathy symptoms, include specific interventions indicated by the manifestations (for example, for abnormal sweating or nocturnal diarrhoea).<br><br>**Diabetic foot problems 1.7.11** For guidance on preventing and managing foot problems in adults with type 2 diabetes, see the NICE guideline on diabetic foot problems.<br><br>**Diabetic foot problems guidance** 1.3.4 When examining the feet of a person with diabetes, remove their shoes, socks, bandages and dressings, and examine both feet for evidence of the following risk factors: Neuropathy (use a 10 g monofilament as part of a foot sensory examination)…<br><br>2.2 Randomised controlled trials are therefore needed to better understand the treatment choices that are available which improve blood glucose control and long-term risks of complications associated with diabetes.
Kidney function	Blood pressure management: Add medications if lifestyle advice does not reduce blood pressure to below 140/80 mmHg (below 130/80 mmHg if there is kidney, eye or cerebrovascular damage). Monitor blood pressure every 1–2 months, and intensify therapy if the person is already on antihypertensive drug treatment, until the blood pressure is consistently below 140/80 mmHg (below 130/80 mmHg if there is kidney, eye or cerebrovascular damage).<br><br>1.4.2 Make changes only if there is poor control or if current drug treatment is not appropriate because of microvascular complications or metabolic problems.<br><br>**1.4.3**: Repeat blood pressure measurements within …2 months if blood pressure is higher than 130/80 mmHg and there is kidney, eye or cerebrovascular damage.

SCORE-IT COS	NICE guideline (text taken from "Key priorities for implementation", "Recommendations" and "Research Recommendations")
	**1.4.4**: Provide lifestyle advice (see section 1.3 in this guideline and the lifestyle interventions section in hypertension in adults [NICE guideline CG127]) if blood pressure is confirmed as being consistently above 140/80 mmHg (or above 130/80 mmHg if there is kidney, eye or cerebrovascular damage).<br><br>Similar conditional statement (…if there is kidney, eye or cerebrovascular damage) in **1.4.5 1.4.6**<br><br>**1.6.10** If adults with type 2 diabetes achieve an HbA1c level that is lower than their target and they are not experiencing hypoglycaemia, encourage them to maintain it. Be aware that there are other possible reasons for a low HbA1c level, for example, deteriorating renal function or sudden weight loss.<br><br>**Diabetic kidney disease 1.7.12** For guidance on managing kidney disease in adults with type 2 diabetes, see the NICE guideline on chronic kidney disease in adults.
Activities of daily living (including those related to personal care; household tasks or community based tasks)	
Body weight	**1.3.4** Dietary advice: Integrate dietary advice with a personalised diabetes management plan, including other aspects of lifestyle modification, such as increasing physical activity and losing weight.<br><br>**1.3.5**: For adults with type 2 diabetes who are overweight, set an initial body weight loss target of 5–10%. Remember that lesser degrees of weight loss may still be of benefit, and that larger degrees of weight loss in the longer term will have advantageous metabolic impact.<br><br>**1.3.10** For recommendations on lifestyle advice, see the NICE guidelines on: preventing excess weight gain, weight management, obesity, physical activity, smoking: brief interventions and referrals, stop smoking services, smoking: harm reduction, and smoking: acute, maternity and mental health services.<br><br>**1.6.10** If adults with type 2 diabetes achieve an HbA1c level that is lower than their target and they are not experiencing hypoglycaemia, encourage them to maintain it. Be aware that there are other possible reasons for a low HbA1c level, for example, deteriorating renal function or sudden weight loss.<br><br>2.1 There is limited understanding of the short- and long-term effects of stopping a therapy and switching to another in terms of diabetes control (HbA1c levels), hypoglycaemic risk, weight gain, and cardiovascular morbidity and mortality<br><br>**1.6.29** Only continue GLP-1 mimetic therapy if the person with type 2 diabetes has had a beneficial metabolic response (a reduction of at least 11 mmol/mol [1.0%] in HbA1c and a weight loss of at least 3% of initial body weight in 6 months).<br><br>**Footnote [9]** Medicines and Healthcare products Regulatory Agency (MHRA) guidance (2011) notes that cases of cardiac failure have been reported when pioglitazone was used in combination with insulin, especially in patients with risk factors for the development of cardiac failure. It advises that if the combination is used, people should be observed for signs and symptoms of heart failure, weight gain, and oedema.
