# Supplementary material for: Core outcome sets through the healthcare ecosystem: the case of type 2 diabetes mellitus
Source: Trials. 2020 Jun 25;21:570. doi: 10.1186/s13063-020-04403-1 (PMC7318375; doi:10.1186/s13063-020-04403-1)
Supplement: Supplementary file 1 — Additional file 1: Supplementary Table 1a. Outcomes in COS for research for T2D (SCORE-IT), COS for routine care (ICHOM set), NICE QS and QI, CPRD and DECIDE trial. Supplementary Table 1b. Outcomes in NICE guidelines. Supplementary Table 1c. Outcomes in FDA guidelines. Supplementary Table 1d. Outcomes in EMA guidelines. [file 13063_2020_4403_MOESM1_ESM.zip › Supplementary Table 1c REVISEDR1.pdf]

Supplementary Table 1c. Outcomes in FDA guidelines

SCORE-IT COS	FDA Guidance for Industry – Type 2 Diabetes Mellitus: Evaluating the Safety of New Drugs for Improving Glycemic Control
Overall survival	
Death from a diabetes related cause such as heart disease	**C. Other Considerations**<br>Adverse CV outcomes remain an important source of morbidity and mortality for patients with type 2 diabetes mellitus. Therefore, sponsors should use rigorous methods for the collection of adverse CV events and assess them by adjudication.
Heart failure	**B. Patient Characteristics in the Development Program**<br>Patients with type 2 diabetes mellitus often have comorbid conditions and/or diabetes-associated complications (e.g. chronic kidney disease, CV disease). Therefore, it is important to evaluate the safety of new drugs to improve glycemic control in the population of patients who will be using the drugs, including a meaningful number of patients with underlying CV disease, chronic kidney disease, and older patients….<br><br>**C. Other Considerations**<br>Adverse CV outcomes remain an important source of morbidity and mortality for patients with type 2 diabetes mellitus. Therefore, sponsors should use rigorous methods for the collection of adverse CV events and assess them by adjudication.
Gangrene or amputation of the leg, foot or toe	**B. Patient Characteristics in the Development Program**<br>Patients with type 2 diabetes mellitus often have comorbid conditions and/or diabetes-associated complications (e.g. chronic kidney disease, CV disease). Therefore, it is important to evaluate the safety of new drugs to improve glycemic control in the population of patients who will be using the drugs, including a meaningful number of patients with underlying CV disease, chronic kidney disease, and older patients….
Hyperglycaemic emergencies (to include diabetic ketoacidosis and hyperosmolar hyperglycaemic state)	**B. Patient Characteristics in the Development Program**<br>Patients with type 2 diabetes mellitus often have comorbid conditions and/or diabetes-associated complications (e.g. chronic kidney disease, CV disease). Therefore, it is important to evaluate the safety of new drugs to improve glycemic control in the population of patients who will be using the drugs, including a meaningful number of patients with underlying CV disease, chronic kidney disease, and older patients….
Hyperglycaemia	**B. Patient Characteristics in the Development Program**<br>Patients with type 2 diabetes mellitus often have comorbid conditions and/or diabetes-associated complications (e.g. chronic kidney disease, CV disease). Therefore, it is important to evaluate the safety of new drugs to improve glycemic control in the population of patients who will be using the drugs, including a meaningful number of patients with underlying CV disease, chronic kidney disease, and older patients….
Hypoglycaemia	**B. Patient Characteristics in the Development Program**<br>Patients with type 2 diabetes mellitus often have comorbid conditions and/or diabetes-associated complications (e.g. chronic kidney disease, CV disease). Therefore, it is important to evaluate the safety of new drugs to improve glycemic control in the population of patients who will be using the drugs, including a meaningful number of patients with underlying CV disease, chronic kidney disease, and older patients….
Cerebrovascular disease (including stroke. subarachnoid haemorrhage, transient ischaemic attack and vascular dementia)	**B. Patient Characteristics in the Development Program**<br>Patients with type 2 diabetes mellitus often have comorbid conditions and/or diabetes-associated complications (e.g. chronic kidney disease, CV disease). Therefore, it is important to evaluate the safety of new drugs to improve glycemic control in the population of patients who will be using the drugs, including a meaningful number of patients with underlying CV disease, chronic kidney disease, and older patients….
Hospital admissions due to diabetes	
Side effects of treatment	**A. Size of the Safety Database**<br>…drugs approved to improve glycemic control in these patients should have well-characterized safety profiles based on shorter term studies, but some safety concerns may only be identified in longer term studies…<br><br>**C. Other Considerations**<br>Sponsors should also consider the following concerning the collection of safety data:<br>Adverse CV outcomes remain an important source of morbidity and mortality for patients with type 2 diabetes mellitus. Therefore, sponsors should use rigorous methods for the collection of adverse CV events and assess them by adjudication.

SCORE-IT COS	FDA Guidance for Industry – Type 2 Diabetes Mellitus: Evaluating the Safety of New Drugs for Improving Glycemic Control
	In some cases, the evaluation of a premarket safety concern may require that a drug development program accrue a minimum number of relevant adverse events to exclude a meaningful degree of risk. Adjudication of these adverse events may also be needed. The Agency expects that situations where the collection of these additional safety data is necessary will be identified and discussed before phase 3 trials are initiated.
Global quality of life (including physical. mental. and social wellbeing)	
Nonfatal myocardial infarction	**B. Patient Characteristics in the Development Program**<br>Patients with type 2 diabetes mellitus often have comorbid conditions and/or diabetes-associated complications (e.g. chronic kidney disease, CV disease). Therefore, it is important to evaluate the safety of new drugs to improve glycemic control in the population of patients who will be using the drugs, including a meaningful number of patients with underlying CV disease, chronic kidney disease, and older patients….<br><br>**C. Other Considerations**<br>Adverse CV outcomes remain an important source of morbidity and mortality for patients with type 2 diabetes mellitus. Therefore, sponsors should use rigorous methods for the collection of adverse CV events and assess them by adjudication.
Visual deterioration or blindness	**B. Patient Characteristics in the Development Program**<br>Patients with type 2 diabetes mellitus often have comorbid conditions and/or diabetes-associated complications (e.g. chronic kidney disease, CV disease). Therefore, it is important to evaluate the safety of new drugs to improve glycemic control in the population of patients who will be using the drugs, including a meaningful number of patients with underlying CV disease, chronic kidney disease, and older patients….
Glycaemic control	**A. Size of the Safety Database**<br>…drugs approved to improve glycemic control in these patients should have well-characterized safety profiles…<br><br>**B. Patient Characteristics in the Development Program**<br>…it is important to evaluate the safety of new drugs to improve glycemic control in the population of patients who will be using the drugs…
Neuropathy (damage to the nerves caused by high glucose. This can lead to tingling and pain or numbness in the feet or legs. It can also affect bowel control; stomach emptying and sexual function)	**B. Patient Characteristics in the Development Program**<br>Patients with type 2 diabetes mellitus often have comorbid conditions and/or diabetes-associated complications (e.g. chronic kidney disease, CV disease). Therefore, it is important to evaluate the safety of new drugs to improve glycemic control in the population of patients who will be using the drugs, including a meaningful number of patients with underlying CV disease, chronic kidney disease, and older patients….
Kidney function	**B. Patient Characteristics in the Development Program**<br>Patients with type 2 diabetes mellitus often have comorbid conditions and/or diabetes-associated complications (e.g. chronic kidney disease, CV disease). Therefore, it is important to evaluate the safety of new drugs to improve glycemic control in the population of patients who will be using the drugs, including a meaningful number of patients with underlying CV disease, chronic kidney disease, and older patients….
Activities of daily living (including those related to personal care; household tasks or community based tasks)	
Body weight	
