# Supplementary material for: Core outcome sets through the healthcare ecosystem: the case of type 2 diabetes mellitus
Source: Trials. 2020 Jun 25;21:570. doi: 10.1186/s13063-020-04403-1 (PMC7318375; doi:10.1186/s13063-020-04403-1)
Supplement: Supplementary file 1 — Additional file 1: Supplementary Table 1a. Outcomes in COS for research for T2D (SCORE-IT), COS for routine care (ICHOM set), NICE QS and QI, CPRD and DECIDE trial. Supplementary Table 1b. Outcomes in NICE guidelines. Supplementary Table 1c. Outcomes in FDA guidelines. Supplementary Table 1d. Outcomes in EMA guidelines. [file 13063_2020_4403_MOESM1_ESM.zip › Supplementary Table 1dR1.pdf]

Supplementary Table 1d. Outcomes in EMA guidelines

SCORE-IT COS	EMA Guideline on clinical investigation of medicinal products in the treatment or prevention of diabetes mellitus
Overall survival	
Death from a diabetes related cause such as heart disease	
Heart failure	4.2.4 Effect on long term complications: Long term complications include macrovascular (coronary, cerebrovascular, and peripheral vascular diseases) and microvascular complications (retinopathy, nephropathy, and neuropathy).<br><br>4.6.3 Cardiovascular safety: It is expected that the drug development program, containing all relevant clinical and non-clinical data, adequately characterizes the cardiovascular safety profile enabling an evaluation of the cardiovascular safety in the marketing authorization application.
Gangrene or amputation of the leg, foot or toe	4.2.4 Effect on long term complications: Long term complications include macrovascular (coronary, cerebrovascular, and peripheral vascular diseases) and microvascular complications (retinopathy, nephropathy, and neuropathy).
Hyperglycaemic emergencies (to include diabetic ketoacidosis and hyperosmolar hyperglycaemic state)	
Hyperglycaemia	
Hypoglycaemia	4.2.2.1 Combined endpoints e.g. reflecting the percentage of patients achieving target HbA1c without hypoglycaemia can be informative as secondary endpoints in some situations but should be prespecified.<br>4.2.2.3 In insulin-treated type 2 diabetic patients, the entire elimination of the need for insulin in a clinically meaningful proportion of patients, or a relevant reduction in insulin dose accompanied by a clinically meaningful improvement in the evolution of body weight or reduction in hypoglycaemic events could be considered as a relevant measure of efficacy, in addition to improvement in or maintenance of HbA1c.<br>4.4.4.1 In the maintenance period the dose(s) of the glucose lowering agent(s) (investigational drug, background therapy, comparator) should be kept stable unless a dose adaption is necessary for safety reasons (e.g. hypoglycaemia).<br>4.4.4.4 Reasons for such consideration may be frequent and especially severe hypoglycaemic events preventing the desired level of glycaemic control or insulin-induced weight gain in already obese patients… Secondary endpoints should, amongst others, include frequency of hypoglycaemia with focus on severe events, change in body weight and in insulin dose and may also include the percentage of patients achieving target HbA1c without hypoglycaemia.<br>4.6.2 Hypoglycaemia: A standardised definition of severe and less severe episodes of hypoglycaemia should be used as defined by Learned Societies to include a set of symptoms and a given level of self-monitored blood glucose... Hypoglycaemia should be confirmed by measuring capillary or plasma glucose levels whenever possible… A detailed analysis of hypoglycaemic episodes noted in clinical trials should be provided<br>5.4.4 Patients should be treated to glycaemic target taking into account limiting adverse effects, particularly hypoglycaemia.<br>5.5.1 Particular attention should be paid to the occurrence of hypoglycaemia and optimal dose titration in these patients.<br>5.5.2 HbA1c is the recommended primary efficacy endpoint. Glycaemic variability and hypoglycaemic episodes are important secondary endpoints<br>5.6.1 Hypoglycaemia A relevant reduction of documented episodes of hypoglycaemia, particularly severe events, if studied in appropriately controlled trials, could support a claim of superiority over the insulin used as comparator provided that this is not achieved with simply allowing HbA1c to rise.<br>5.6.5 Frequent hypoglycaemic as well as hyperglycaemic episodes may impair cognitive development and should be avoided.
Cerebrovascular disease (including stroke. subarachnoid	4.2.4. Effect on long term complications: Long term complications include macrovascular (coronary, cerebrovascular, and peripheral vascular diseases) and microvascular complications (retinopathy, nephropathy, and neuropathy).

SCORE-IT COS	EMA Guideline on clinical investigation of medicinal products in the treatment or prevention of diabetes mellitus
haemorrhage, transient ischaemic attack and vascular dementia)	4.4.4.2 …beneficial effects on micro and/or macrovascular endpoints and a well characterized safety profile (including data on long term safety) should be documented before a first line monotherapy indication would be considered approvable.
Hospital admissions due to diabetes	
Side effects of treatment	4.4.2 If there are pharmacologically active metabolites, the contribution to therapeutic and/or toxic effects should be discussed.<br>4.4.4.2 …beneficial effects on micro and/or macrovascular endpoints and a well characterized safety profile (including data on long term safety) should be documented before a first line monotherapy indication would be considered approvable.<br>4.5.2 Therefore it is not recommended that studies in children/adolescents are initiated before sufficient safety and efficacy data from adult trials are available.<br>4.6.1 Special efforts should be made to capture potential adverse events that are characteristic of the mechanism of action and the pharmacodynamic properties of the class of products being investigated.<br>5.4.4 Patients should be treated to glycaemic target taking into account limiting adverse effects, particularly hypoglycaemia.<br>5.6.2. Local reactions / toxicity: Pain at the injection site and any type of local reaction should be carefully monitored, particularly in patients on long term treatment.
Global quality of life (including physical. mental. and social wellbeing)	*4.2.5 The use of disease-specific patient-reported outcomes for diabetes is recommended as it may reveal important information on how a treatment affects quality-of-life*.
Nonfatal myocardial infarction	4.2.4 Effect on long term complications: Long term complications include macrovascular (coronary, cerebrovascular, and peripheral vascular diseases) and microvascular complications (retinopathy, nephropathy, and neuropathy).<br><br>4.6.3 Cardiovascular safety: It is expected that the drug development program, containing all relevant clinical and non-clinical data, adequately characterizes the cardiovascular safety profile enabling an evaluation of the cardiovascular safety in the marketing authorization application.
Visual deterioration or blindness	4.2.4. Effect on long term complications: Long term complications include macrovascular (coronary, cerebrovascular, and peripheral vascular diseases) and microvascular complications (retinopathy, nephropathy, and neuropathy).<br>5.6.3 In case of higher affinity to the IGF-1 receptor of insulin analogues compared to human insulin, it is recommended that fundus photographs are taken during long term trials to detect possible retinal adverse events.
Glycaemic control	4.2.2. Measures of glycaemic control (4.2.2.1. Haemoglobin A1c; 4.2.2.2. Plasma glucose; 4.2.2.3. Insulin parameters)<br>4.3.1 Glycaemic control (4.3.1.1. Haemoglobin A1c, 4.3.1.2. Plasma glucose, 4.3.1.3. Insulin sensitivity/Beta cell function)<br>4.4.3 FPG should be the primary evaluation criterion in dose-ranging studies of 8-12 weeks duration. Serum fructosamine can also be used as an endpoint in short term studies. However HbA1c should always be the primary evaluation criterion in dose-ranging studies of ≥12 weeks duration.<br>4.4.4.1 Superiority of the new agent over placebo in at least one monotherapy study of no less than 3 months duration, which could be a dose-ranging, phase II study using HbA1c as the primary endpoint… The primary endpoint should be HbA1c while secondary endpoints should include other measures of glycaemic control as well as the effect on other cardiovascular risk factors<br>4.4.4.4 Reasons for such consideration may be frequent and especially severe hypoglycaemic events preventing the desired level of glycaemic control or insulin-induced weight gain in already obese patients… The primary objective of the study should be to demonstrate that the test drug is superior to placebo in HbA1c reduction… Secondary endpoints should, amongst others, include frequency of hypoglycaemia with focus on severe events, change in body weight and in insulin dose and may also include the percentage of patients achieving target HbA1c without hypoglycaemia.<br>4.5.2 In principle the change in HbA1c from baseline to at least 12 weeks versus the control may be acceptable as a primary endpoint<br>5.5.2 HbA1c is the recommended primary efficacy endpoint. Glycaemic variability and hypoglycaemic episodes are important secondary endpoints

SCORE-IT COS	EMA Guideline on clinical investigation of medicinal products in the treatment or prevention of diabetes mellitus
	5.6.1 A relevant reduction of documented episodes of hypoglycaemia, particularly severe events, if studied in appropriately controlled trials, could support a claim of superiority over the insulin used as comparator provided that this is not achieved with simply allowing HbA1c to rise.
Neuropathy (damage to the nerves caused by high glucose. This can lead to tingling and pain or numbness in the feet or legs. It can also affect bowel control; stomach emptying and sexual function)	4.2.4. Effect on long term complications: Long term complications include macrovascular (coronary, cerebrovascular, and peripheral vascular 261 diseases) and microvascular complications (retinopathy, nephropathy, and neuropathy).
Kidney function	4.2.4. Effect on long term complications: Long term complications include macrovascular (coronary, cerebrovascular, and peripheral vascular diseases) and microvascular complications (retinopathy, nephropathy, and neuropathy).<br>4.4.1 It should be taken into consideration that factors such as delayed gastric emptying and gastrointestinal transit time or altered renal function can be expected to complicate drug absorption and disposition in a significant number of type 2 diabetic patients.
Activities of daily living (including those related to personal care; household tasks or community based tasks)	4.3.2. Patient-reported outcomes: The inclusion of patient-reported outcomes to assess the treatment burden and impact on daily life, diabetes management, compliance and cognition is recommended. In this case it is important that the questionnaires or scales are validated for use in the setting of diabetes.
Body weight	4.2.2.3 In insulin-treated type 2 diabetic patients, the entire elimination of the need for insulin in a clinically meaningful proportion of patients, or a relevant reduction in insulin dose accompanied by a clinically meaningful improvement in the evolution of body weight or reduction in hypoglycaemic events could be considered as a relevant measure of efficacy, in addition to improvement in or maintenance of HbA1c.<br>4.2.3 Short- and long-term effects of the tested product on serum lipids (LDL and HDL cholesterol, triglycerides), body weight and other parameters associated with body composition (e.g. waist circumference) as well as blood pressure and heart rate should be documented.<br>4.4.4.4 Reasons for such consideration may be frequent and especially severe hypoglycaemic events preventing the desired level of glycaemic control or insulin-induced weight gain in already obese patients. Overall, the most frequently used combination is insulin plus metformin… Secondary endpoints should, amongst others, include frequency of hypoglycaemia with focus on severe events, change in body weight and in insulin dose and may also include the percentage of patients achieving target HbA1c without hypoglycaemia.<br>5.3.1 Weight gain is frequent in diabetic patients trying to implement intensive glucose control. The evolution of body weight will also be taken into account in the global evaluation of the efficacy and safety, particularly in type 2 diabetic patients.
